# Supplementary material for: Nanomechanical DNA resonators for sensing and structural analysis of DNA-ligand complexes
Source: Nat Commun. 2019 Apr 12;10:1690. doi: 10.1038/s41467-019-09612-0 (PMC6461617; doi:10.1038/s41467-019-09612-0)
Supplement: Supplementary file 1 — Supplementary Information [file 41467_2019_9612_MOESM1_ESM.pdf]

SUPPLEMENTARY INFORMATION

Nanomechanical DNA resonators for sensing and structural analysis of DNA-ligand complexes

Stefano Stassi[1,§], Monica Marini[1,§], Marco Allione[2], Sergei Lopatin[3], Domenico Marson[4], Erik Laurini[4], Sabrina Pricl[4], Candido Fabrizio Pirri[1], Carlo Ricciardi[1,], Enzo Di Fabrizio[2,*]*

[1]Dipartimento di Scienza Applicata e Tecnologia, Politecnico di Torino, Corso Duca Degli Abruzzi, 24, 10129 Torino, Italy

[2]Physical Science and Engineering and BESE Divisions, King Abdullah University of Science and Technology, Thuwal 23955-6900, Saudi Arabia

[3]Imaging and Characterization Core Lab, King Abdullah University of Science and Technology, Thuwal 23955-6900, Kingdom of Saudi Arabia

[4]Molecular Biology and Nanotechnology Laboratory (MolBNL@UniTS) – DEA, University of Trieste, Piazzale Europa 1,34127 Trieste, Italy

Corresponding Author: carlo.ricciardi@polito.it, Enzo.DiFabrizio@KAUST.EDU.SA

[§]These authors contributed equally

SUPPLEMENTARY FIGURES

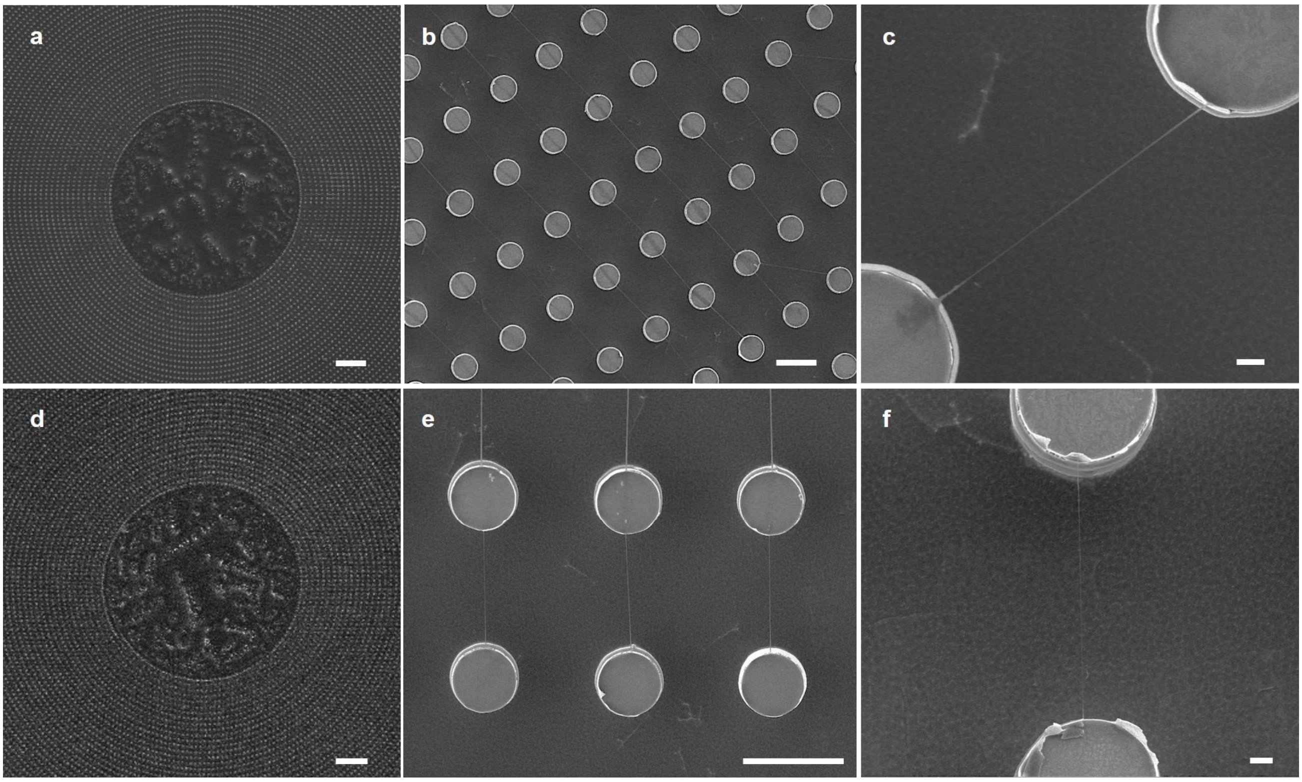

Supplementary Figure 1 | DNA/GelRed and DNA/YOYO–1 SEM images. (a) Low magnification image of the DNA/GelRed complex solution after complete evaporation over SHS. The salts removed from the bundles are concentrated over an area of 500 µm diameter of a device constituted by micro-fabricated cylindrical pillars (height 10 µm, diameter 6 µm, gap 12 µm) (b) DNA/GelRed intercalated bundles are suspended between pillars showing a behaviour comparable to the non intercalated bundles. (c) The DNA/GelRed bundles are homogenously suspended on the device and are linked to the edge of adjacent micro-pillars, coherently with previously reported data. Analogous observations can be reported for the DNA/YOYO–1 complexes (images d-f). Scale bars correspond to 100 µm for (a,d), 10 µm for (b,e) and 1 µm for (e,f).

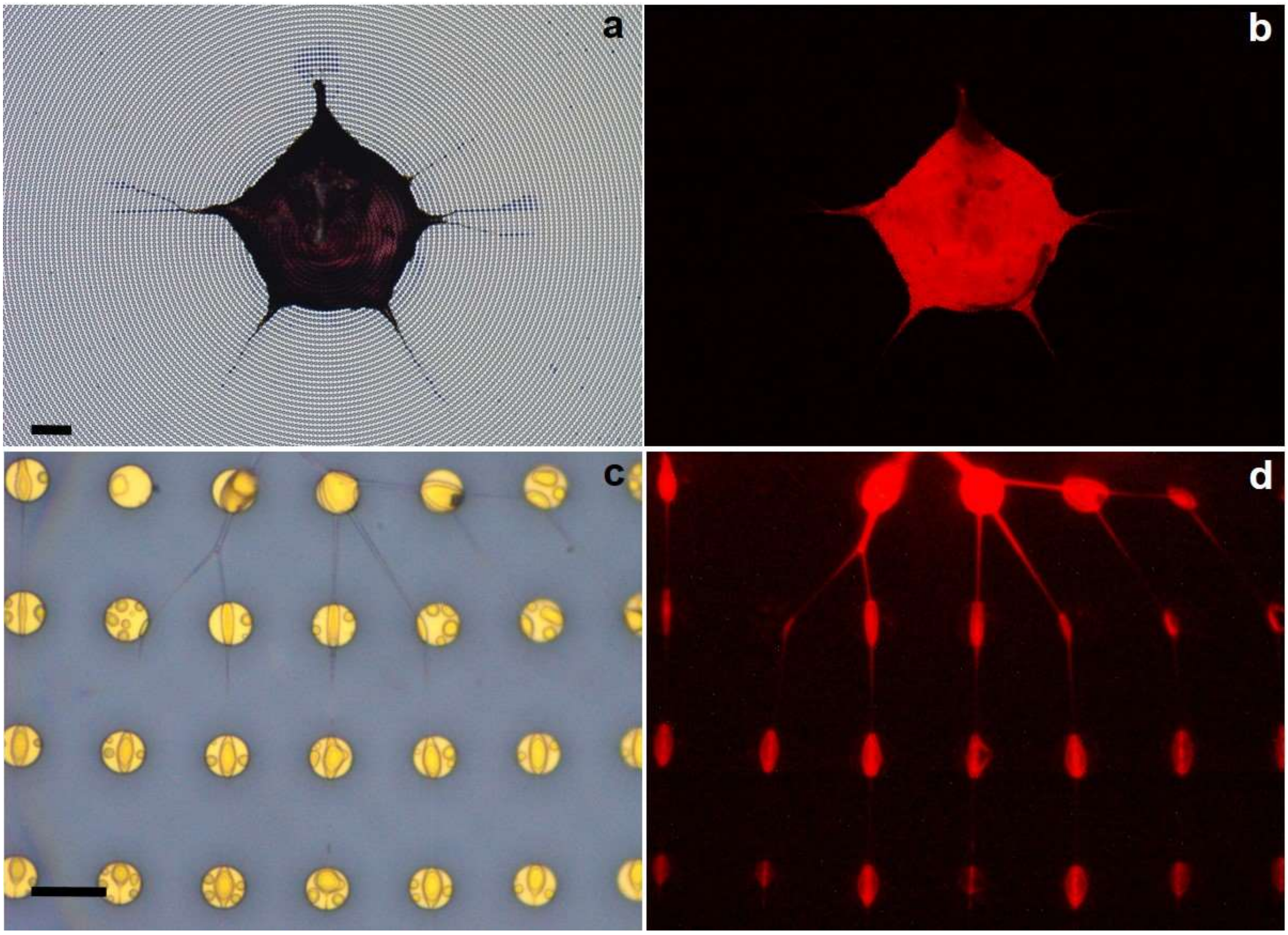

Supplementary Figure 2 | DNA/GelRed optical images. (a,c) Bright field optical images of the suspended intercalated DNA bundles. (b,d) Correspondent images acquired using an excitation of 540/25 nm (DM 565, BA 605/55). The scale bar corresponds to 100 µm in (a) and to 10 µm in (c).

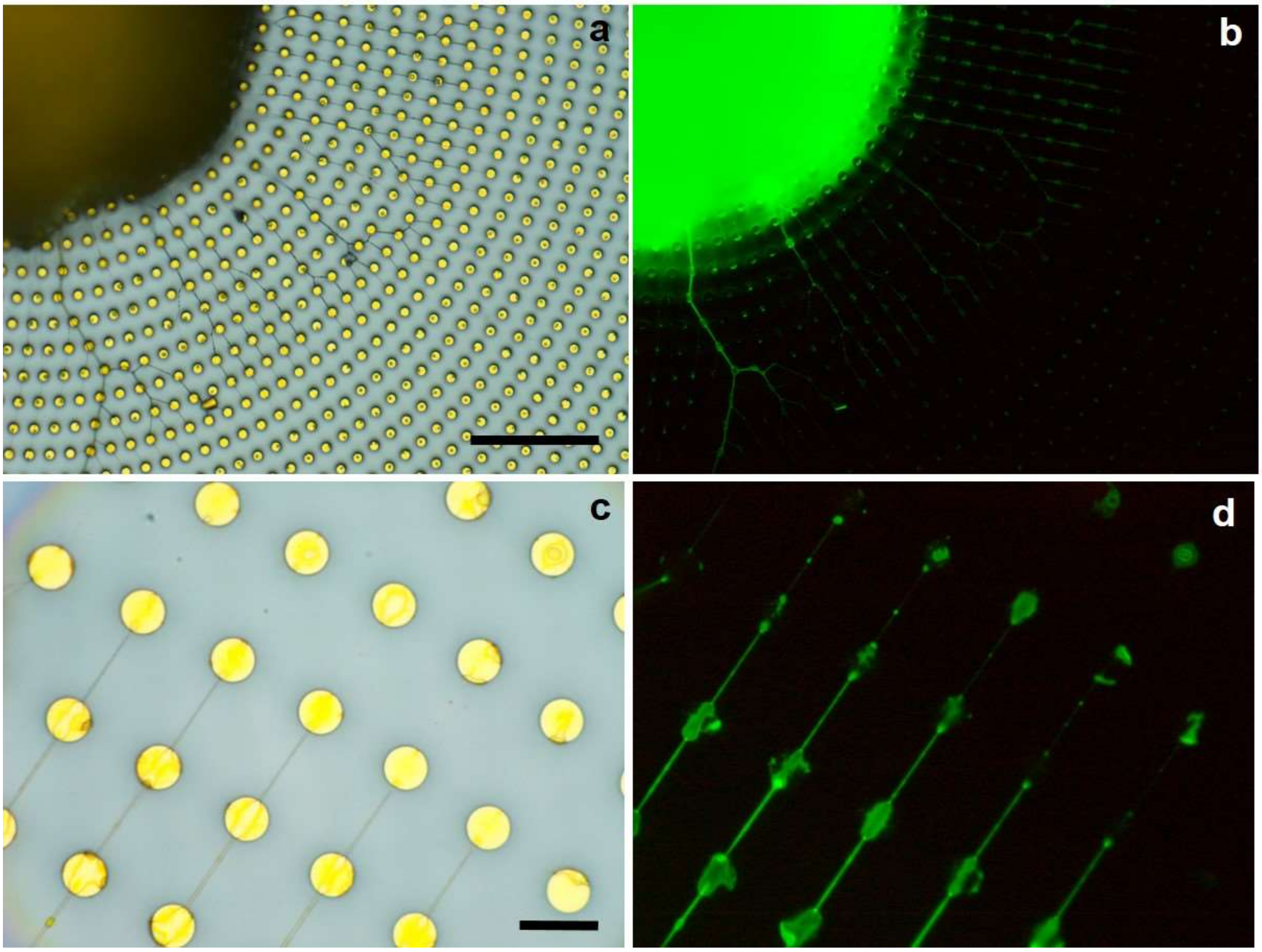

Supplementary Figure 3 | DNA/YOYO–1 optical images. (a,c) Bright field optical images of the suspended DNA bundles intercalated with YOYO-1. (b,d) On the right, the correspondent images acquired using an excitation of 465–495 nm. The scale bar corresponds to 100 μm in (a) and to 10 μm in (c).

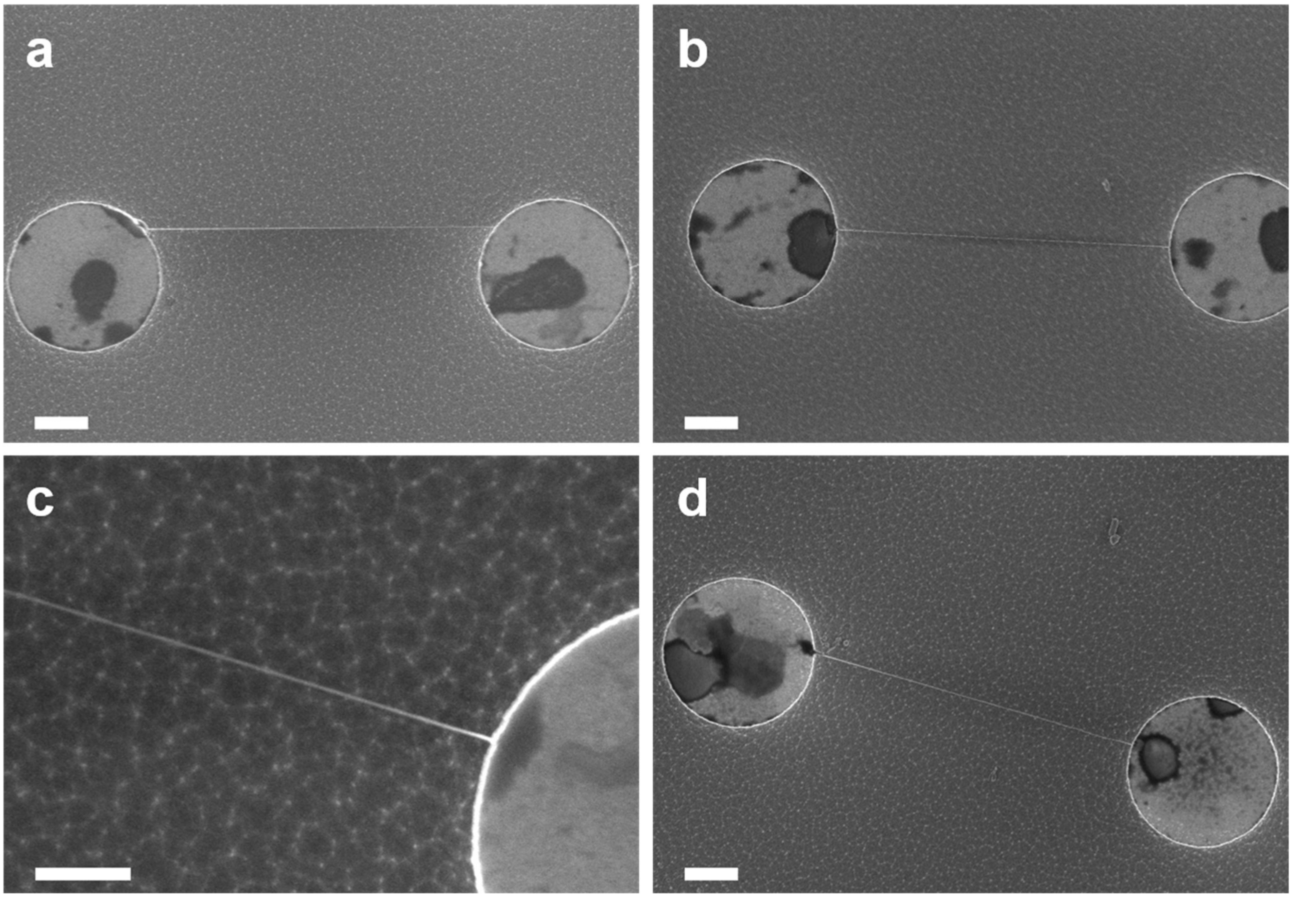

Supplementary Figure 4 | SEM images of DNA bundles intercalated with cisplatin. (a-d) SEM images of different DNA bundles intercalated with cisplatin showing the stability and homogeneity of the structures. The scale bars correspond to 2 μm, except for (c), which corresponds to 1 μm.

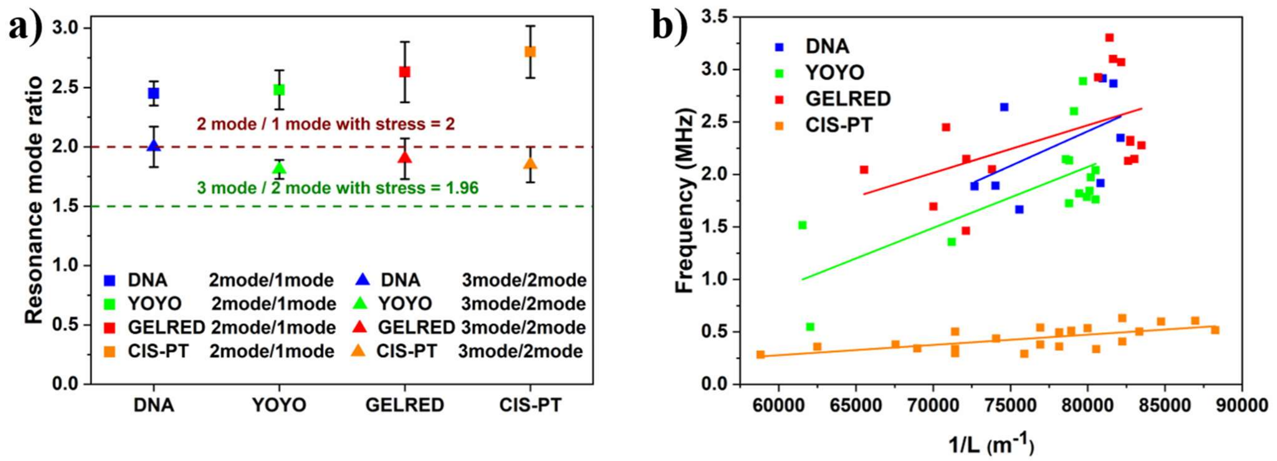

Supplementary Figure 5 | Tensile stress analysis on the DNA bundles. (a) Resonance mode ratio evaluated on bare DNA resonators and DNA intercalated with YOYO–1, GelRed and CisPt. Dashed lines represent the theoretical value of the ratio between the resonance modes for a clamped-clamped beam in a regime dominated by stress. Error bars were computed from the standard deviation of 25 measurements. Source data are provided as a raw data file. (b) Frequency of the fundamental resonance mode as a function of the inverse of the resonator length. Tentative linear fits of the data are reported for each kind of DNA sample. The plots do not show a good agreement with a linear trend for each type of DNA bundles confirming that the resonators are not in a stress dominating regime, but the resonance frequency depends on the flexural rigidity of the structure.

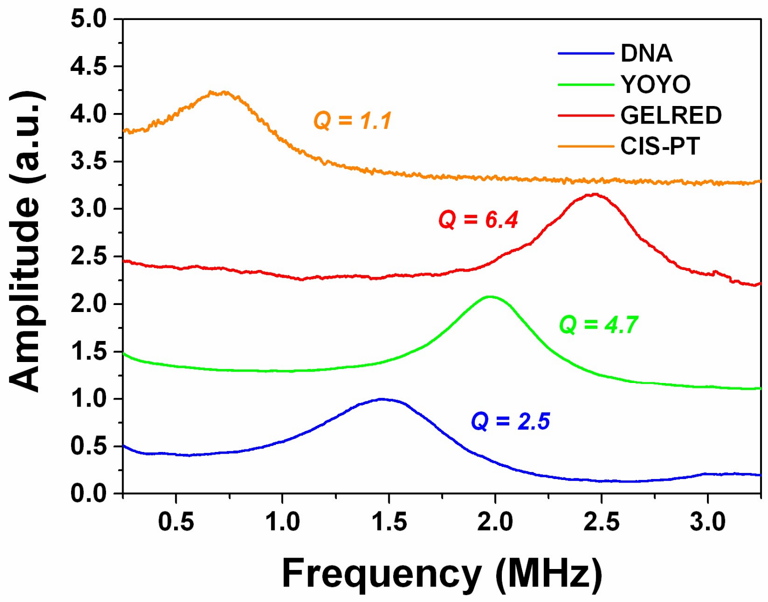

Supplementary Figure 6 | First resonance mode of the pristine and intercalated DNA resonators measured in air. The graph reports the vibration spectra of the four different DNA resonator measured in air environment for the evaluation of the quality factor. The amplitudes of the spectra were normalized and vertically distributed for a better clarity. Since the values of Q factor depend on the rigidity of the structure, a similar correspondence with the trend of the Young's modulus was found in the quality factor values for the bare DNA resonators and the intercalated DNA ones. The Q factor is higher in resonators intercalated with fluorescent molecules, YOYO−1 and GelRed, with respect to the bare DNA bundle, while decreases in the intercalated CisPt resonator.

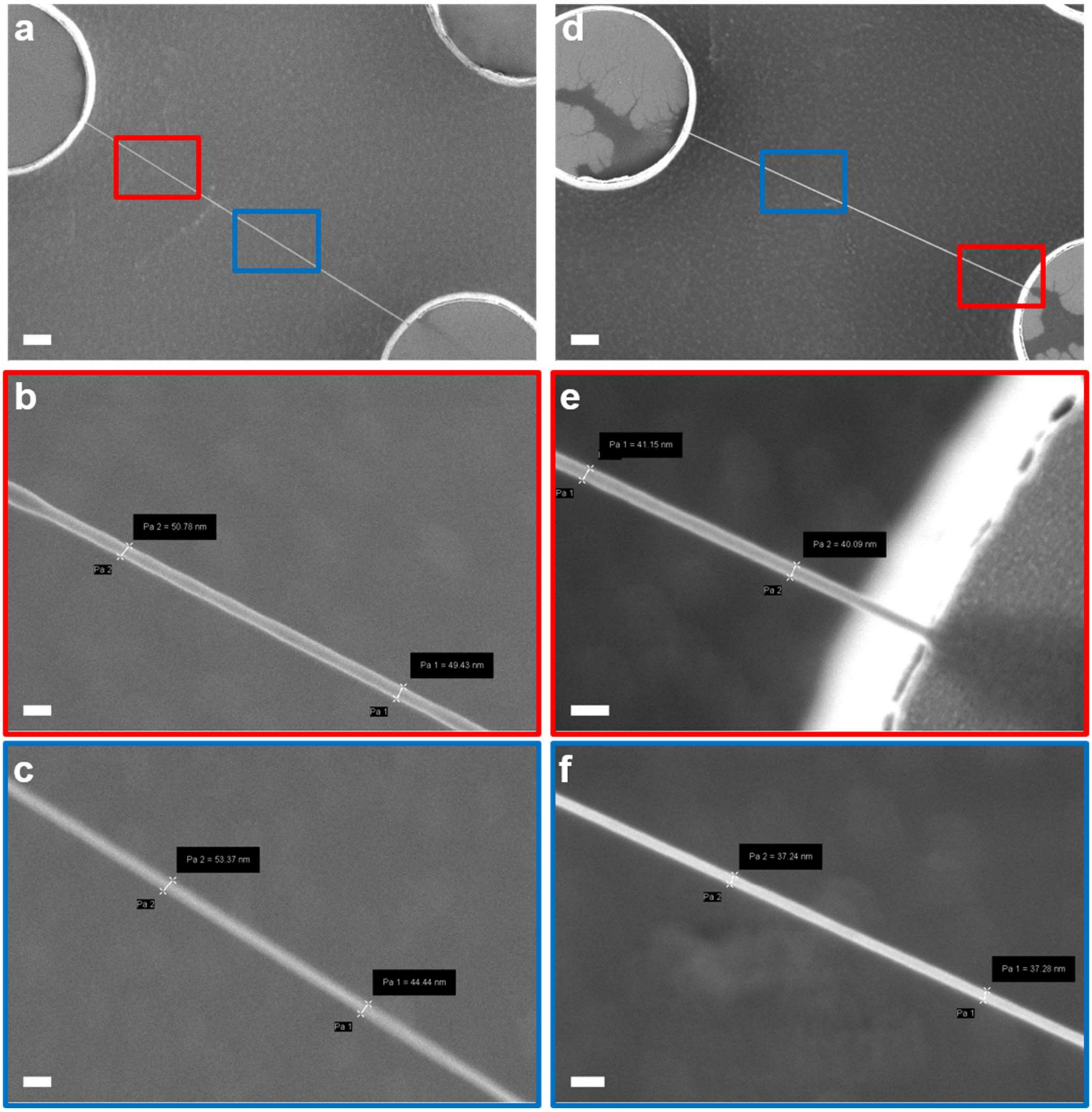

Supplementary Figure 7 | Diameter analysis of the DNA bundles. SEM images of two different DNA bundles (a-c and d-f) with reported the measurements of the diameter. The coloured boxes in (a) and (d) underline the area where the other images (b,c and e,f) were taken. The scale bar corresponds to 100 nm except for (a,d) which corresponds to 1 µm.

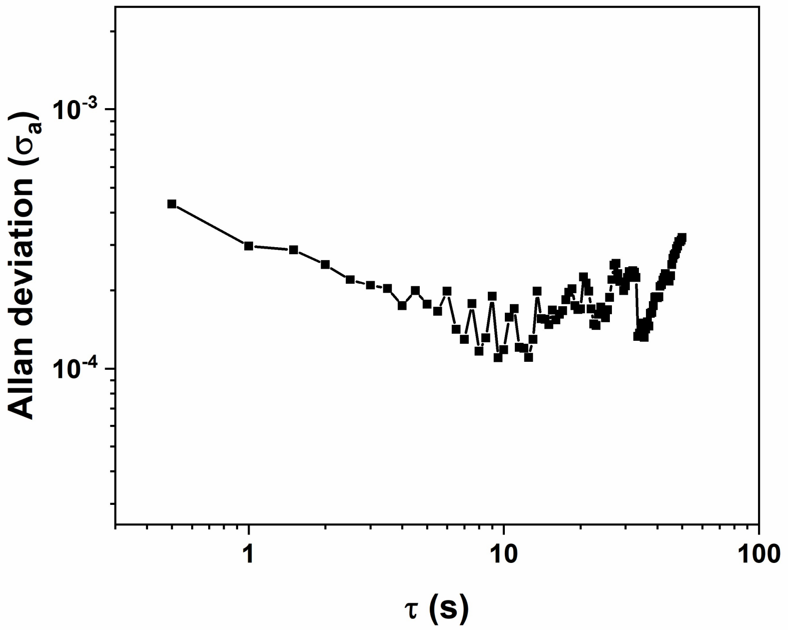

Supplementary Figure 8 | Allan deviation of the DNA resonator. The graph reports the frequency stability of a DNA bundles measured in air environment. The minimum of the Allan deviation was used for the evaluation of the limit of detection of the DNA resonators.

SUPPLEMENTARY TABLES

Supplementary Table 1 | R^2 coefficients of the fit of the DNA resonance frequencies. The table reports the R^2 coefficients computed from the linear fit of the resonance frequency values of the different DNA bundles plotted as a function of the ratio of the radius over the square of the length (R/L^2) and of the inverse of the resonator length (1/L). The linear fits are reported in Figure 6b and in Supplementary Figure 5b

	**R^2 of linear fit for Freq vs R/L^2**	**R^2 of linear fit for Freq vs 1/L**
**DNA**	0.717	0.158
**YOYO**	0.966	0.448
**GELRED**	0.696	0.225
**CIS-PT**	0.692	0.495

SUPPLEMENTARY NOTES

Supplementary Note 1

In the following are reported the analyses performed to determine whether, the suspended DNA bundles can be modeled as clamped-clamped beams in a regime dominated by stress, or flexural rigidity. The ratio among the first three resonance modes was studied (Figure 6a in the manuscript and Supplementary Figure 5a) and the dependence of the resonance frequency over R/L^2 (flexural rigidity regime, Figure 6b) or 1/L (stress regime, Supplementary Figure 5b) was reported. For an easier understanding of the fitting procedure of the measured resonance frequencies, we reported in Supplementary Table 1 the R^2 coefficients computed from the linear fitting of both curves of Figure 6b and in Supplementary Figure 5b. Even if for some fitting the R^2 is not very high, the values related to flexural rigidity are always remarkably higher with respect to the stress regime one. Therefore, all the analyses confirmed that the resonator composed by pristine or intercalated DNA are in a regime dominated by flexural rigidity and that at first approximation, the internal stress component can be neglected.

Supplementary Note 2

The diameter of the DNA bundles was estimated with a SEM analysis. Each DNA resonator was observed with SEM and the diameter was estimated with a post-processing analysis, measuring the dimension over different points of the bundles. The bundles were quite homogeneous over the length with an average variability around 7%. In this percentage, we included residual salts and hydration shell variation. Most of them present a slightly higher diameter close to the clamping and a narrowing in the centre. Some images of the bundles are reported as example in Supplementary Figure 7.

The preparation technique based on the super-hydrophobic approach (described in detail in the Methods section of the manuscript) returned bundles with variable diameters over the whole chip. DNA bundles with smaller diameter were obtained at the border of the samples, while getting closer to the centre of the sample and of the deposited droplet, the bundle diameter increases. In this work, we evaluated bundles with different diameter from 30 to 100 nm to study in details the mechanical properties of the DNA and understand if the resonators were in a flexural or stress dominated regime.

For the evaluation of Young's modulus of each single bundle type, bare DNA or intercalated, we considered the average values. Indeed, the value of Young's modulus for each DNA sample family was extracted from the distribution of the values obtained by measuring several different bundles. Then, the error was computed as standard deviation of the same distribution.

Supplementary Note 3

The frequency noise of the DNA resonator was computed by performing different consecutive measurements of the thermal noise spectrum of the bundles. Each measurement takes up to 0.5 s. For each spectrum the resonance frequency was evaluated with a Lorentzian fit of the peak. The resonance frequency values were then used to compute the Allan deviation σ_a of the resonator in the integration time τ as:

$$\sigma_a = \sqrt{\frac{1}{2(N-1)} \sum_{i=2}^{N} \left(\frac{\bar{f}_i - \bar{f}_{i-1}}{f_0} \right)^2} \quad (1)$$

where $\bar{f}_i$ is the time average of the frequency measurement in the ith time interval of time τ, N is the total number of intervals, and f_0 is the mean resonance frequency value calculated over the whole duration of the measurement. The plot of the Allan deviation of the DNA resonator is reported in Supplementary Figure 8.

The frequency stability of the resonator to be used as the minimum detectable resonance frequency change $\delta f_{\min}$ was extracted from the minimum of the Allan deviation. This value was then used to compute the limit of detection (LOD) as:

$$\text{LOD} = \delta m_{\min} = \frac{\delta f_{\min}}{S} \quad (2)$$

From the analysis of the vibrational response of the DNA resonators resulted a theoretical sensitivity ranging from 4.2 to 9 Hz/ag depending on the length and diameter of the bundle. Considering the frequency stability of the resonator, the limit of detection of the DNA resonators is between 22 and 48 ag.
