# Supplementary material for: Nanomechanical DNA resonators for sensing and structural analysis of DNA-ligand complexes
Source: Nat Commun. 2019 Apr 12;10:1690. doi: 10.1038/s41467-019-09612-0 (PMC6461617; doi:10.1038/s41467-019-09612-0)
Supplement: Supplementary file 3 — Description of Additional Supplementary Files [file 41467_2019_9612_MOESM3_ESM.pdf]

Description of Additional Supplementary Files

File name: Supplementary Movie 1

Description: Movies of Steered Molecular Dynamic simulations on pristine DNA. The movies report the SMD simulation performed with the highest stress on a pristine DNA filament. DNA is portrayed in blue as both transparent solvent accessible surface area and cartoon representation. On the bottom left of the movie is reported the elongation of the DNA over simulated time. In the graphs, the abscissa shows the time of simulation (from 0 to 20 ns), while the ordinate shows the DNA elongation (in the range 0 to 120 Å). The graphs develop over the movie time, in synchronized with the portrayed DNA elongation.

File name: Supplementary Movie 2

Description: Movies of Steered Molecular Dynamic simulations on DNA intercalated with CisPT. The movies report the SMD simulation performed with the highest stress on a DNA filament intercalated with cisplatinum. DNA is portrayed in blue as both transparent solvent accessible surface area and cartoon representation, while CisPt is portrayed as Van Der Waals spheres in orange. On the bottom left of each movie is reported the elongation of the DNA over simulated time. In the graphs, the abscissa shows the time of simulation (from 0 to 20 ns), while the ordinate shows the DNA elongation (in the range 0 to 160 Å). The graphs develop over the movie time, in sync with the portrayed DNA elongation.

File name: Supplementary Movie 3

Description: Movies of Steered Molecular Dynamic simulations on DNA intercalated with YOYO-1. The movies report the SMD simulation performed with the highest stress on a DNA filament intercalated with YOYO-1. DNA is portrayed in blue as both transparent solvent accessible surface area and cartoon representation, while YOYO is portrayed as Van Der Waals spheres in green. On the bottom left of each movie is reported the elongation of the DNA over simulated time. In the graphs, the abscissa shows the time of simulation (from 0 to 20 ns), while the ordinate shows the DNA elongation (in the range 0 to 160 Å). The graphs develop over the movie time, in sync with the portrayed DNA elongation.
